# Nance-Horan Syndrome: characterization of dental, clinical and molecular features in three new families

**DOI:** 10.1186/s12903-023-03029-4

**Published:** 2023-05-23

**Authors:** Yeliz Guven, Hilal Piril Saracoglu, Sermin Dicle Aksakal, Tugba Kalayci, Umut Altunoglu, Zehra Oya Uyguner, Serpil Eraslan, Esra Borklu, Hulya Kayserili

**Affiliations:** 1 Department of Pedodontics, Faculty of Dentistry, Istanbul University, Vezneciler, Istanbul, Turkey; 2 Graduate School of Heath Sciences, Koc University, Sarıyer, Istanbul, Turkey; 3 Department of Medical Genetics, Istanbul Medical Faculty, Istanbul University, Istanbul, Turkey; 4 Department of Medical Genetics, Koc University School of Medicine (KUSoM), Sarıyer, Istanbul, Turkey; 5 Genetic Diseases Evaluation Center, Koc University Hospital, Zeytinburnu, Istanbul, Turkey

**Keywords:** Nance–Horan syndrome, Cataracts and teeth anomalies, Hutchinson teeth, Bud-shaped molars, Screwdriver shaped incisors, Supernumerary teeth

## Abstract

**Background:**

Nance–Horan syndrome (NHS; MIM 302,350) is an extremely rare X-linked dominant disease characterized by ocular and dental anomalies, intellectual disability, and facial dysmorphic features.

**Case presentation:**

We report on five affected males and three carrier females from three unrelated NHS families. In Family 1, index (P1) showing bilateral cataracts, iris heterochromia, microcornea, mild intellectual disability, and dental findings including Hutchinson incisors, supernumerary teeth, bud-shaped molars received clinical diagnosis of NHS and targeted *NHS* gene sequencing revealed a novel pathogenic variant, c.2416 C > T; p.(Gln806*). In Family 2, index (P2) presenting with global developmental delay, microphthalmia, cataracts, and ventricular septal defect underwent SNP array testing and a novel deletion encompassing 22 genes including the *NHS* gene was detected. In Family 3, two half-brothers (P3 and P4) and maternal uncle (P5) had congenital cataracts and mild to moderate intellectual deficiency. P3 also had autistic and psychobehavioral features. Dental findings included notched incisors, bud-shaped permanent molars, and supernumerary molars. Duo-WES analysis on half-brothers showed a hemizygous novel deletion, c.1867delC; p.(Gln623ArgfsTer26).

**Conclusions:**

Dental professionals can be the first-line specialists involved in the diagnosis of NHS due to its distinct dental findings. Our findings broaden the spectrum of genetic etiopathogenesis associated with NHS and aim to raise awareness among dental professionals.

**Supplementary Information:**

The online version contains supplementary material available at 10.1186/s12903-023-03029-4.

## Background

Nance–Horan syndrome (NHS) (MIM 302,350) is an extremely rare X-linked disorder characterized by congenital cataracts, dental anomalies, and facial dysmorphic features. Nance et al. and Horan and Billson were the first to describe the disease features simultaneously in 1974 [1, 2]. A dense congenital nuclear cataract is the main ocular finding of NHS syndrome and leads to decreased visual acuity at an early age. Microcornea, microphthalmia, nystagmus, and strabismus may also be observed [3–5]. The most common dental abnormalities in males are screwdriver-shaped incisors, supernumerary teeth, diastema, and tapered premolar/molar cusps [6, 7]. Facial dysmorphic features are long narrow face, prominent and bulbous nose, and anteverted pinnae [7, 8]. Intellectual disability is reported in 30% of affected males [9]. Lateral brachymetacarpalia [8] and congenital cardiac defects [10] have also been described. Carrier females display less severe clinical findings such as lens opacities around the posterior Y-sutures with little or no loss of vision, mild facial dysmorphism, and dental abnormalities [1, 11].

NHS is caused by mutations in 10 coding exons of the *NHS* gene spanning 650 kb in Xp22.13. The *NHS* gene encodes at least five different isoforms as a result of alternative splicing. The NHS protein is expressed in the brain, retina and lens, tooth primordia, craniofacial mesenchyme, and heart. The *NHS* gene is highly conserved among vertebrates [12]. Phenotypic variability has been reported in several cases with different pathogenic variants.

To date, 55 different disease-causing *NHS* variants have been reported in HGMD database (HGMD professional; Sep 20, 2022). The majority are missense/nonsense (31%) and small deletion (29%) variants. Additionally, four regulatory or splicing substitutions, seven small insertions, seven gross deletions/insertions, and four complex rearrangements have been reported [13].

Congenital cataracts, the major finding of NHS, is an accompanying feature in a large number of syndromes, thus presenting a challenge for early and prompt diagnosis. On the other hand, dental findings present in almost all cases of NHS are highly distinctive for the diagnosis. The present study reports on three different NHS families diagnosed with three different molecular approaches. We further discuss the diagnostic value of orodental manifestations in five NHS-affected males and two carrier females, along with accompanying clinical features and molecular findings.

## Case presentation

Three families with five affected males were included in the study. Informed consent was obtained from all patients and their guardians by local clinicians both for molecular analysis and the release of photographs. The study protocol was approved by the Ethics Committees of the Istanbul Medical Faculty at Istanbul University and Koç University. Peripheral blood samples from the patients and their mothers were obtained for molecular studies.

**Fig. 1:**
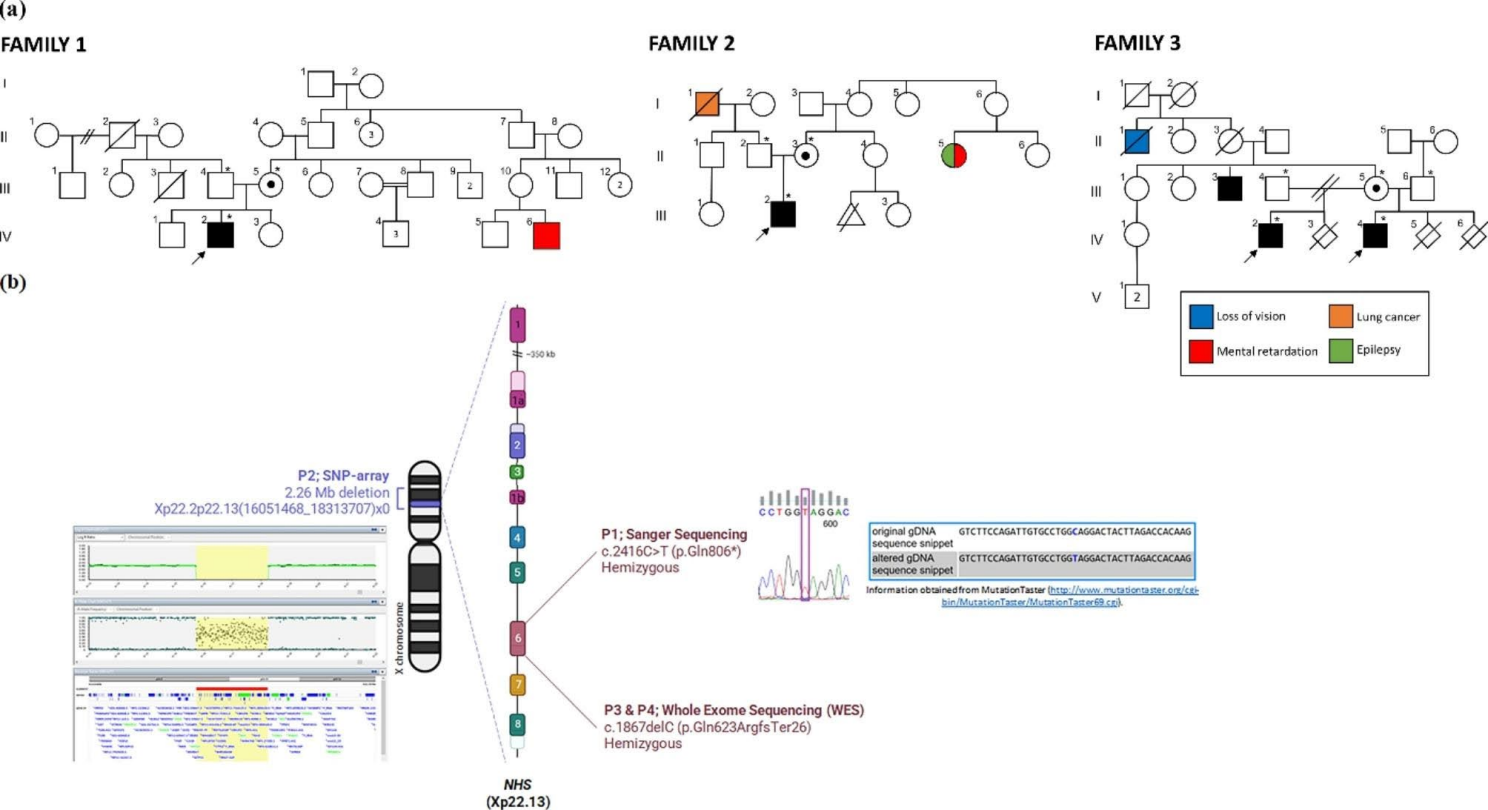
(**a**) Pedigrees of three NHS families. Probands are indicated by arrows. Affected individuals are indicated by filled-in symbols. Obligate carriers are represented by a central black dot. (**b**) Ideogram of X chromosome indicating the localization of the *NHS* gene on Xp22.13. Schematic organization of the *NHS* gene, exons in scale and three different molecular diagnostic techniques are shown. P1: Electropherogram of the *NHS* gene exon 6. P2: SNP array analysis. P3 and P4: WES analysis

A detailed clinical description of the cases is provided in Supplementary Table. Pedigrees are shown in Fig. 1a. Diagnostic molecular approaches and the variations identified are shown on schematic drawing of the *NHS* gene (Fig. 1b).

### Family 1 (Patient 1)

**Fig. 2:**
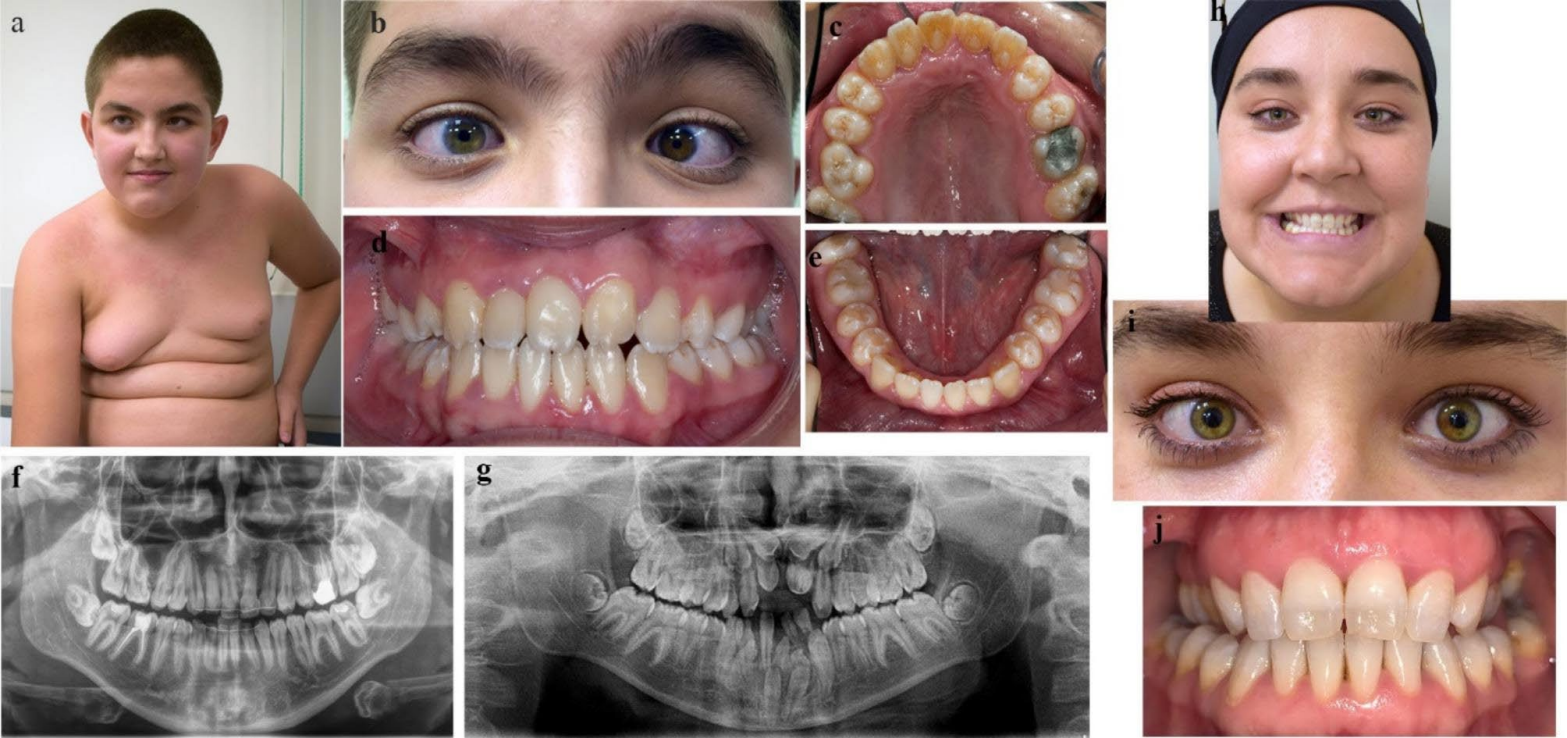
Clinical and radiographic images of affected proband (a-g) and carrier mother (h-j) from Family 1. (**a**) Note the bilateral gynecomastia and mild dysmorphism with short philtrum, short palpebral fissures, and anteverted pinnae (**b**) Close-up view of eyes showing bilateral rotatory nystagmus, strabismus on the left eye, and heterochromia. (**c-e**) Intraoral images at 15 years old demonstrating Hutchinson incisors, bud-shaped molars, talon cusps in maxillary and mandibular incisors, and localized hypomineralised areas of enamel in the incisal edges and cusp tips. (**f**) Panoramic radiograph at 15 years old (**g**) Panoramic radiograph at 10 years old showing the supernumerary teeth in anterior maxilla and mandible. (**h-i**) Facial and eye close-up view of mother. Note the partial heterochromia of both eyes. (**j**) Intraoral image shows tapered maxillary central incisors and increased translucency of enamel in nearly half of the incisors’ crowns

The proband (P1; IV:2) was a 10-year-old boy who presented to the Department of Pedodontics, Faculty of Dentistry of Istanbul University, due to missing anterior teeth. Radiographic examination exhibited supernumerary incisors in the maxilla and mandible inhibiting the eruption of the incisors and early eruption of second molars (Fig. 2g). Bud-shaped molars were noted on intraoral examination. Follow-up treatment of two years duration included removal of supernumerary teeth and orthodontic traction of unerupted maxillary teeth till permanent dentition was complete. When anterior teeth become visible in the oral cavity, additional findings, including Hutchinson incisors, talon cusps in maxillary and mandibular incisors, and localized hypomineralised areas of enamel in the incisal edges and cusp tips, were observed (Fig. 2c-f).

The patient was born full term via repeat cesarean section with normal birth weight and length to non-consanguineous parents. He was noted to have bilateral congenital cataracts, bilateral rotatory nystagmus, and strabismus on the left eye. He also had heterochromia as an additional finding (Fig. 2a-b). He underwent bilateral cataract surgery at the age of one and had been operated upon for glaucoma multiple times thereafter. He had mild intellectual disability and had received special education since the age of seven. He was diagnosed with attention deficit hyperactivity disorder (ADHD). Extraoral examination revealed mild dysmorphic features such as short philtrum, short palpebral fissures, and anteverted pinnae. The mother presented with partial heterochromia of both eyes and was followed up for early-onset cataracts between the ages of 34 to 37. She exhibited mild dental findings. The upper central incisors were slightly tapered, and coronal half of their crowns had increased translucency (Fig. 2h-j).

Distinct dental findings led to the clinical diagnosis of NHS, and Sanger sequencing was planned to identify the pathogenic variant. Sanger sequencing of the *NHS* gene (GRCh38.p12: ENST00000380060.7;NM198270.4) was performed with gene-specific primers for nine exons, comprising the coding regions with min.25 bp from the 5’ and 3’ intronic region. All primers were designed using NCBI Primer-Blast(ref) (sequences can be provided upon request). A BigDye® Terminator v3.1 Cycle Sequencing Kit (ThermoFisher, USA) was used according to the manufacturer’s instructions, and capillary electrophoresis was performed on a 3500XL Genetic Analyzer (ABI-Hitachi, USA). The *.ab1 files were analyzed using a FinchTV free chromatogram viewer (Geospiza, Inc., USA).

A novel nonsense variant, c.2416 C > T; p.(Gln806*), in exon 6 was identified in the *NHS* gene and it was predicted to be likely pathogenic by *in silico* analyses (Polyphen2 and Mutation Taster2). The mother (III:5) was heterozygous for the pathogenic variant, whereas the unaffected son had wild type allele.

### Family 2 (Patient 2)

**Fig. 3:**
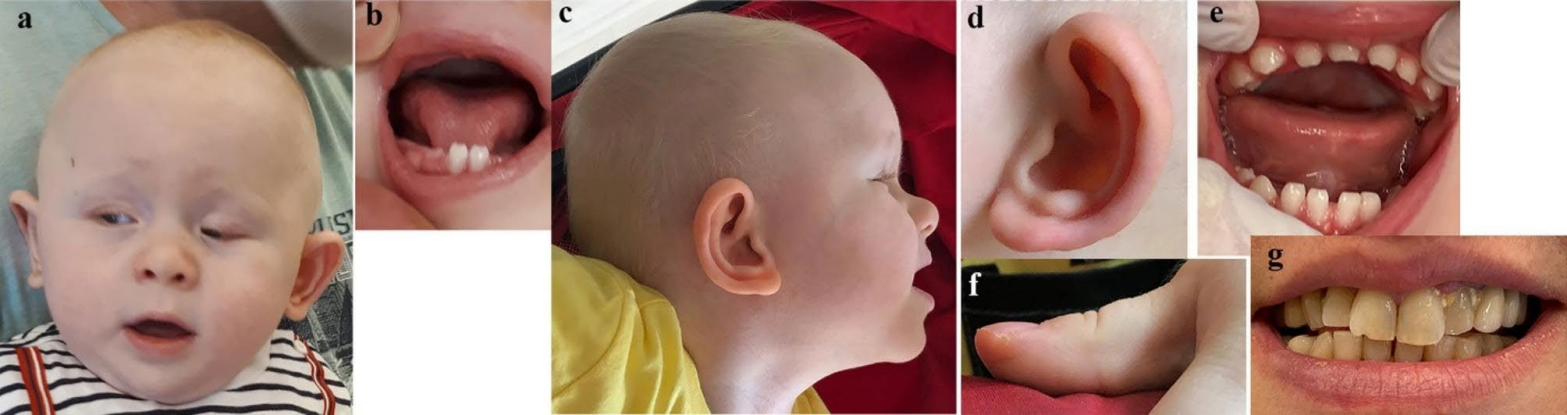
Clinical images of affected proband (**a-f**) and carrier mother (**g**) from Family 2. (a-b) Facial and intraoral view at 6 months of age showing microphthalmia and mild dysmorphic features, including short and narrow palpebral fissures and lower eyelid entropion. Note the screwdriver-shaped lower incisors. (**c-d**) Lateral view at 21 months of age demonstrating depressed nasal bridge, anteverted nares, and distinct ears. (**e**) Note the abnormal crease on the tip of the right thumb. (**f**) Intraoral view at 21 months of age showing screwdriver shaped incisors (**g**) Intraoral image of mother. Note the notch on the incisal edge of the upper right incisor

The proband (P2; III:2), a 6-month-old male, was referred to the Genetic Diseases Evaluation Center of Koc University Hospital due to bilateral congenital cataracts, microphthalmia, and a ventricular septal defect. He was the first child of a non-consanguineous couple and was born at full term with a normal birth weight and length after an uneventful pregnancy. He had undergone bilateral cataract surgery twice, at three and five months of age. He also had microcornea and nystagmus-like movements. He showed global developmental delay and mild dysmorphic features, including short and narrow palpebral fissures, lower eyelid entropion, depressed nasal bridge, and anteverted nares. Bilateral fifth finger clinodactyly, an abnormal crease on the tip of the right thumb, and retractile testicles were noted (Fig. 3a,c,d,f).

The presence of congenital anomalies accompanying neurodevelopmental delay prompted the clinical geneticist to follow an algorithmic evaluation for the diagnosis, and 300 K SNP-array was carried out. The SNP-array adopted was a 300 K HumanCytoSNP-12v2-1 (Illumina Inc., San Diego, CA). Genomic DNA was processed according to the supplier’s instructions (Illumina Inc). Arrays were scanned using an i-Scan instrument (Illumina Inc., San Diego, CA). Illumina KaryoStudio and BlueFuse Multi were the software used to computationally determine the breakpoints, with default settings. Genomic positions were based on the UCSC February 2009 human reference sequence (hg19) (NCBI build 37 reference sequence assembly).

SNP-array analysis revealed a 2.26 Mb hemizygous deletion in the short arm of chromosome X (band p22), between base pairs of 16,051,468 − 18,313,707. (Xp22.2p22.13(16051468_18313707)x0). The deletion region encompassed 22 genes, *GRPR, MAGEB17, RPL6P30, RN7SL658P, CTPS2, MIR548AM, S100G, SYAP1, RNU7-56P, TXLNG, RBBP7, RNU4-6P, REPS2, CBX1P2, CBX1P4*, ***NHS***, *MIR4768, NHS-AS1, SCML1, RAI1, BEND2*, and *SCML2.* SNP-array 300 K analysis on the maternal DNA sample revealed the same deletion at heterozygote state.

The patient was referred for dental examination after the molecular diagnosis of NHS at 19 months of age. At the first dental examination, only lower incisors were present, and they had a screwdriver shape (Fig. 3b).

At two years of age, psychomotor developmental delay was more prominent, and tactile sensitivity was noted. The patient spoke with single words and was able to walk independently for only two to three steps. On intraoral examination, the patient had completely erupted 18 teeth. The primary incisors were screwdriver shaped and the edges of upper primary incisors were crescent shaped (Fig. 3e). The mother had a notch on the incisal edge of upper right incisor that is suggestive of Hutchinson tooth (Fig. 3g).

### Family 3 (Patients 3-4-5)

The proband (P3; IV:2), a 36-year-old male, was referred to the Medical Genetics Department of Istanbul Medicine Faculty due to ocular anomalies and autistic features. He was the first child of non*-*consanguineous parents, born at full term with a normal birth weight and length. He was diagnosed with bilateral congenital cataracts at 3 months of age and was operated upon. He spoke two-word sentences at 42 to 48 months and walked at 36 months. He had unilateral cryptorchidism and underwent gonadectomy at the age of eight.

The patient had moderate intellectual disability and had received special education. He was diagnosed with autism spectrum disorder. Psychiatric evaluation revealed obsessive–compulsive traits, self-mutilation, aggressive behavior patterns (biting and pinching), and sleep disturbance. Ocular anomalies included microcornea, retinal dystrophy, bilateral nystagmus, left corneal opacity, and exotropia. Glaucoma had developed secondary to cataract surgery.

**Fig. 4:**
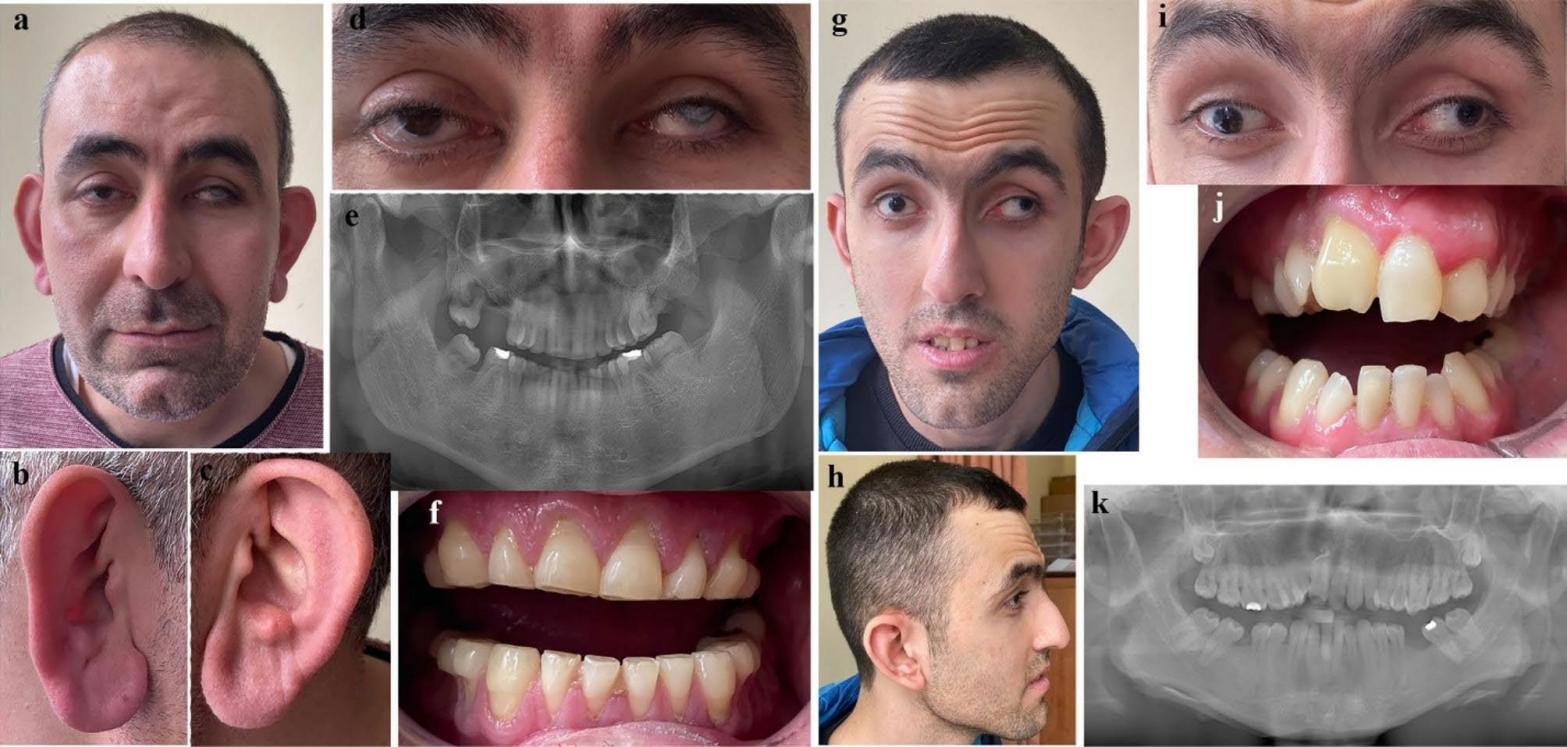
Clinical and radiographic images of affected proband (**a-f**) and his affected maternal half-brother (**g-k**) from Family 3. (**a-c**) Facial and ear close up views showing mild dysmorphic features, including prominent nasal bridge, overfolded and underfolded helix, and prominent and large lobulated ears. (**d**) Eye close up view demonstrating left corneal opacity and exotropia. (**e, f**) Intraoral and radiographic images demonstrating severe attrition of all teeth and rounded-shaped molars. (**g, h**) Frontal and lateral views showing mild dysmorphic features, including a triangular-shaped face with mid-face hypoplasia, arched eyebrows, low-hanging columella, low-set posteriorly rotated right ear, and a prominent left one. (**i**) Note the small irises and left exotropia (**j, k**) Intraoral and radiographic images demonstrating screwdriver-shaped incisors, rounded-shaped molars, and supernumerary molars

Extraoral examination revealed mild dysmorphic features, including prominent nasal bridge, overfolded and underfolded helix, and prominent and large lobulated ears (Fig. 4a-d). Intraoral examination demonstrated severe attrition of all teeth and rounded-shaped molars. Incisal edges of the anterior teeth were extensively worn; hence it was not possible to evaluate for the presence of Hutchinson teeth (Fig. 4e-f).

The proband’s maternal half-brother (P4; IV:4) was 23 years of age at the time of examination. He was born to non-*consanguineous parents* by caesarian section at 34 weeks of gestation. At 2 months of age, he had been operated upon due to cataracts on both eyes. He had mild intellectual disability and attended a mainstream school. Psychiatric examination demonstrated mild obsessive behavior and sleep disturbance. Ocular findings included left exotropia and post-op glaucoma. He had mild dysmorphic features, including a triangular-shaped face with mid-face hypoplasia, arched eyebrows, small irises, low-hanging columella, low-set posteriorly rotated right ear, and a prominent left one (Fig. 4g-i). Intraoral examination revealed a high-arched and narrow palate, notched incisors, rounded-shaped permanent molars, and supernumerary molars (Fig. 4j-k). Supernumerary teeth on the anterior maxilla had been surgically removed at 15 years of age.

The mother (III:5) had normal intelligence and did not have any dental findings suggestive of NHS. According to pedigree analysis, the proband’s maternal uncle (III:3) had bilateral vision loss and behavioral disturbances. He opted not to attend the clinics for further evaluation and molecular testing.

Two half-brothers and their maternal uncle’s similarly affected state was considered suggestive for X-linked recessive inheritance. The presence of congenital cataracts, intellectual disability, and behavioral disturbances in affected males prompted the clinical geneticist to perform whole-exome sequencing (WES) to determine the genetic etiopathogenesis. Whole-exome sequencing was performed with an Ion AmpliSeq Exome Kit on an Ion Torrent S5 XL (Thermo Fisher Scientific). Quality metrics were assessed from Torrent Suite Software v5.12 (Thermo Fisher Scientific). A bioinformatics pipeline was followed according to the plug-in Torrent Variant Caller (Thermo Fisher Scientific).

A hemizygous novel deletion was identified in exon 6 of the *NHS* gene, leading to a frameshift at codon 623, creating a stop codon 26 nucleotides downstream (c.1867delC [p.(Gln623ArgfsTer26)]). This variant was confirmed by Sanger sequencing on both affected half-siblings and was shown at heterozygous state in their mother, III-5.

## Discussion and conclusions

We here report on an ultra-rare syndrome, NHS, in three Turkish families with five affected males who were diagnosed with three different molecular testing approaches.

NHS is an X-linked syndrome with almost 70 reported cases in the literature to date. Some affected individuals may go unrecognized or even misdiagnosed due to the reduced penetrance and variable expressivity that lead to NHS underdiagnosis. The presence of subtle non-ocular manifestations could easily be overlooked thus the true incidence of NHS in the general population cannot be determined [14].

In family 1, the pediatric dentist referred the patient to the genetics outpatient clinics for further evaluation due to the presence of distinct dental features accompanying ocular findings and intellectual disability. Because NHS was the preliminary clinical diagnosis due to the prominent dental and clinical findings, Sanger sequencing of the *NHS* gene was planned. In family 3, the siblings had bilateral cataracts, varying degree of intellectual disability, and neurobehavioral problems ranging from autism to mild obsessions. Although the pedigree was suggestive for X-linked recessive inheritance, there was no clinical handle to achieve clinical diagnosis. The presence of cataracts and intellectual disability could be observed in several syndromes, therefore, the option to perform WES was considered a priority.

**Fig. 5:**
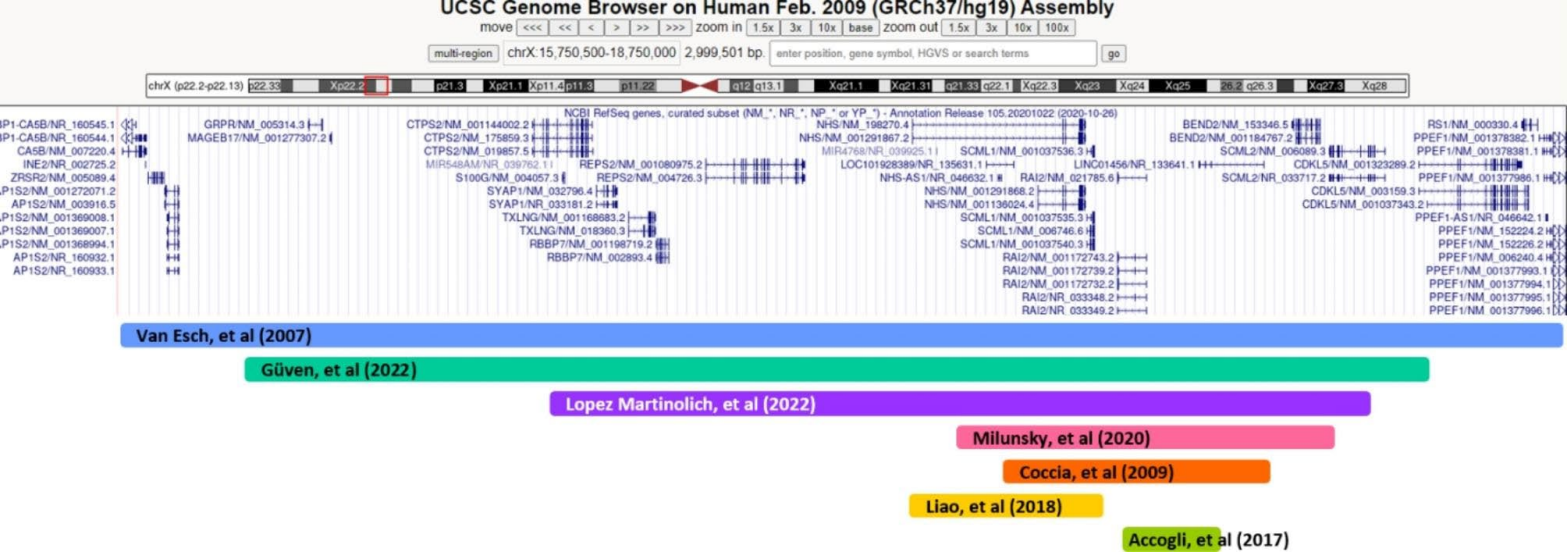
Overlap of the gross deletions encompassing *NHS* reported so far. P2 is the second largest reported deletion in this region that has been detected by SNP-array analysis

In family 2, clinical diagnosis was a challenge because when the patient was consulted at 6 months of age, his teeth had not erupted, and the distinct, pivotal feature of NHS was not, therefore, present. He had developmental delay accompanying congenital cataracts; hence, a diagnostic algorithmic approach was applied, and 300SNP array was performed. The deleted region encompasses 22 genes, including *GRPR, MAGEB17, RPL6P30, RN7SL658P, CTPS2, MIR548AM, S100G, SYAP1, RNU7-56P, TXLNG, RBBP7, RNU4-6P, REPS2, CBX1P2, CBX1P4, NHS, MIR4768, NHS-AS1, SCML1, RAI1, BEND2*, and *SCML2*. The only known disease-associated gene in this region is *NHS*. This is the second largest interstitial deletion reported for NHS syndrome (Fig. 5).

Van Esch et al. 2007 found the largest 2.8 Mb deletion at Xp22 comprising 16 genes in a boy with severe encephalopathy, congenital cataracts, and tetralogy of Fallot [15]. Their reported deleted region entirely overlaps with the deleted region in our patient plus the *CDKL5* gene, which was the causality for encephalopathy in their patient. They proposed that *RAI2* can be associated with the cardiac anomalies considering the fact that reduced retinoic acid signaling results in early cardiac defects in mammalian embryos and various animal models. On the other hand, Liao et al. 2011 identified a 0.92 Mb microdeletion at Xp22.13 encompassing *REPS2, NHS, SCML1*, and *RAI2* genes in two brothers with typical features of NHS with no additional findings [16]. A family described by Coccia et al. 2009 had a deletion of 0.9 Mb comprising the large region of the *NHS* gene from exons 2 to 8 and entire *SCML1, RAI2*, and *CXorf20 genes* [10]. This family also showed typical features of NHS and had no cardiac anomalies. Accoglia et al. 2017 reported another 170.6 kb microdeletion involving the *NHS, SCLML1* and *RAI2* genes in a boy with NHS, with no cardiac malformations [17]. A recent study reporting on a male with congenital cataracts and non-obstructive azoospermia revealed a 721 kb deletion at Xp22.13 encompassing *NHS*, *SCML1* and *RAI2* with no cardiac anomalies [18]. The most recent report by Lopez Martinolich et al. 2022 is on a 19-year-old boy with 1.83-Mb microdeletion at Xp22.2p22.13 encompassing *NHS, CTPS2, S100G, TXLNG, RBBP7, REPS2, SCML1, RAI2*, and *SCML2* genes. The case had multiple eye malformations along with neurological, skeletal and dysmorphic findings and only dental finding reported was screwdriver blade-shaped incisors. In addition to previously reported NHS features, Lopez Martinolich et al. 2022 mentioned that their case had undescended testicles and sensorineural hearing loss with lack of congenital heart defects [19]. In summary, the absence of congenital cardiac anomalies in all seven patients with deletions of *SCML1*, and *RAI2* genes in common, suggest that cardiac anomalies observed in P2 of the cohort and in Van Esch and colleagues’ study may not be related to *SCML1* and/or *RAI2*. Milunsky et al. 2020 also suggested that *SCML1* deletion in a male is highly likely to be associated with azoospermia [18]. Thus, the absence of *SCML1* gene in P2 may require a follow-up for possible reproductive issues.

The most prominent and early-onset manifestation of NHS is the presence of bilateral congenital cataracts. Congenital cataracts represent a genetically heterogeneous disease that is predominantly inherited as an autosomal dominant trait but could also be inherited as autosomal recessive or X linked. More than 100 genes are implicated in its etiology [20]. Nance–Horan syndrome is one of the few syndromes with cataracts that is inherited in X-linked manner [21, 22]. Therefore, in children with congenital bilateral cataracts, whenever the inheritance pattern is suggestive for X linked, Nance–Horan syndrome should be listed in the differential diagnosis. In the present study, four affected males had congenital bilateral cataracts and received cataracts surgery within the first year of life. Earlier studies have shown that 50% of NHS patients exhibited glaucoma secondary to cataract surgery [8, 23, 24]. Three affected males in the present study have developed glaucoma after cataract surgery. The fourth case, P4, did not exhibit glaucoma either in childhood or thereafter. Other than congenital cataracts and glaucoma, our patients also showed other common ocular findings of NHS, including microcornea, strabismus, and nystagmus.

Intellectual disability is a major component of NHS and has been reported in about 30% of affected patients [25]. In this study, all NHS patients had intellectual disability ranging from mild to moderate. This observation is consistent with Gjorup and co-workers’ report demonstrating intellectual disability in all seven NHS patients [26]. Additionally, autism spectrum disorder observed in P3 has also been noted in a limited number of patients to date [14, 16, 26, 27].

The most common dental finding of NHS is the incisors with slightly tapered crowns, which are also defined as “screwdriver-shaped” incisors [26, 28]. These tapering teeth are commonly presented with notched or crescent-shaped incisal edges similar to Hutchinson’s incisors that are characteristic of congenital syphilis [1]. Although screwdriver-shaped incisors have also been associated with several other syndromes, such as Cockayne syndrome, Kabuki syndrome, and Ectodermal dysplasia [29–31], Hutchinson’s incisors have only been linked with NHS other than congenital syphilis. Therefore, Hutchinson’s incisors in either primary or permanent dentition is a specific feature of NHS and is of substantial importance in the definitive clinical diagnosis of the syndrome. In the present study, screwdriver-shaped incisors with notched or crescent shaped edges were observed in three out of four males.

Another dental finding reported is Moon’s molar or bud molars which is used to describe the permanent premolars and molars with more rounded cusps and a narrower occlusal area relative to the bulging outline of the crown. Although the term “Mulberry molar” is still frequently used interchangeably with the term “Moon’s molar”, the distinction between both terms has already been discussed by a number of researchers [26, 32, 33]. Mulberry molars were originally defined as molar teeth having multiple rounded rudimentary enamel cusps with marked hypoplastic defects. In some studies, the presence of an additional cusp in the central fossa with the globular appearance of the crown is defined as a “mulberry molar” [34, 35]. All affected males in our study (other than the 24-month-old male who had only primary teeth), showed bud-shaped permanent molars. Additionally, P1 also had hypoplastic pits through the buccal ridges.

The presence of supernumerary teeth has been reported in 65% of NHS male patients [23]. In the present study, two out of four affected males had supernumerary teeth. All supernumerary teeth of P1 were located both in the anterior maxilla and in the anterior mandible. P4 showed supernumerary teeth in the retromolar area of both sides of the mandible, which is a very rare localization area for supernumeraries. He also had supernumerary teeth in the anterior maxilla that were removed surgically during his adolescent years. Other dental findings reported include tooth agenesis [21, 23], taurodontism [34], and pulp stones [35]. None of the patients reported herein showed taurodontism and pulp stones. P4 had missing right lateral incisor at the time of examination when he was 23 years old. However, we could not confirm if this missing lateral was congenitally missing or accidentally extracted during the removal of supernumerary teeth.

In family 3, although the two brothers carried the same NHS variant, their clinical and dental findings were quite different. Whereas clinical findings were more severe in the elder brother, the dental findings were milder. This observation may motivate researchers to investigate the underlying mechanisms of clinical heterogeneity, particularly those working in developmental dental research area.

In P1, upper and lower second molars which normally begins to erupt at 12–14 years old were fully erupted at 10 years of age. The apices were completely closed at 12 years old. The presence of early eruption in other males could not be assessed. On examination, P2 was only 24-month-old and no permanent teeth had yet erupted. P3 and P4 were in adult ages and the information regarding the actual timing of individual tooth eruption could not be obtained. Early eruption in permanent teeth is not as common as delayed tooth eruption and can be seen in overweight children or those with hyperthyroidism and uncontrolled diabetes [36]. The body mass index of P1 was 31.2 kg/m2 plots at the 99th percentile for his age which denotes obesity. Therefore, premature eruption of permanent teeth in this case could be attributed to obesity, not the syndrome itself.

Our findings not only broaden the spectrum of genetic etiopathogenesis associated with NHS but also highlight the importance of dental professionals to be made aware of the features. Dental professional could be the first-line specialist involved in the diagnosis of NHS since dental findings are highly distinctive for this very rare syndrome.

## Electronic supplementary material

Below is the link to the electronic supplementary material.

Supplementary Material 1

## Data Availability

The datasets used and/or analysed during the current study are available from the corresponding author on reasonable request.
