# Supplementary material for: Nance-Horan Syndrome: characterization of dental, clinical and molecular features in three new families
Source: BMC Oral Health. 2023 May 23;23:314. doi: 10.1186/s12903-023-03029-4 (PMC10204325; doi:10.1186/s12903-023-03029-4)
Supplement: Supplementary file 1 — Supplementary Material 1 [file 12903_2023_3029_MOESM1_ESM.docx]

**Table 1**. Clinical features of NHS affected males and one manifesting female.

| **Family No** | **Family 1** | | **Family 2** |  | **Family 3** | |
| --- | --- | --- | --- | --- | --- | --- |
| **Patient** | **P1** | **Mother of P1** | **P2** | **P3** | **P4** | **P5** |
| Gender | M | F | M | M | M | M |
| Age (at examination) | 15 y/o | 37 y/o | 6 m/o | 36 y/o | 23 y/o | N/A |
| Eyes | Bilateral cataracts, nystagmus microcornea strabismus, heterochromia | Mild cataracts, partial heterochromia | Cataracts, microphthalmia, microcornea, nystagmus-like eye movements | Bilateral cataracts, microcornea, retinal dystrophy, bilateral nystagmus, left corneal opacity, and exotropia | Bilateral cataracts, left exotropia, iris coloboma | Total vision loss |
| Teeth | Supernumerary teeth, Hutchinson incisors, bud shaped molars, talon cusps, early eruption | Tapered incisors | Hutchinson incisors | Bud shaped molars | Supernumerary teeth, Hutchinson incisors, bud shaped molars | N/A |
| Neurologic and Psychiatric findings | Mild intellectual disability | - | Global developmental delay | Moderate intellectual disability, autism, obsessive–compulsive traits, self-mutilation, aggressive behavior patterns (biting and pinching), and sleep disturbance | Mild intellectual disability, mild obsessive behavior and sleep disturbance | Behavioral abnormalities (extent is unknown) |
| Dysmorphic features | Short philtrum, short palpebral fissures, and anteverted pinnae | - | Short and narrow palpebral fissures, lower eyelid entropion, depressed nasal bridge, and anteverted nares | Prominent nasal bridge, overfolded and underfolded helix, prominent and large lobulated ears | Triangular shaped face with mid face hypoplasia, arched eyebrows, small irides, low-hanging columella, low-set posteriorly rotated right ear and a prominent left one | N/A |
| Other | Gynecomastia | - | VSD, rectractable testis, bilateral fifth finger clinodactyly abnormal crease on the tip of the right thumb | - | - | N/A |
